# 3D high-resolution anorectal manometry in patients with perianal fistulas: comparison with 3D-anal ultrasound

**DOI:** 10.1186/s12876-018-0770-6

**Published:** 2018-04-04

**Authors:** Richelle J. F. Felt-Bersma, Maarten S. Vlietstra, Paul F. Vollebregt, Ingrid J. M. Han-Geurts, Vera Rempe-Sorm, Grietje J. H. Vander Mijnsbrugge, Charlotte B. H. Molenaar

**Affiliations:** 1 Department of Gastroenterology and Hepatology, VU University Medical Centre, Amsterdam, The Netherlands; 2 Proctos Clinic, Bilthoven, The Netherlands

**Keywords:** Anorectal manometry, 3D-HRAM, Endoanal ultrasound, 3D-EUS, Anal fistula

## Abstract

**Background:**

Perianal fistula surgery can damage the anal sphincters which may cause faecal incontinence. By measuring regional pressures, 3D-HRAM potentially provides better guidance for surgical strategy in patients with perianal fistulas. The aim was to measure regional anal pressures with 3D-HRAM and to compare these with 3D-EUS findings in patients with perianal fistulas.

**Methods:**

Consecutive patients with active perianal fistulas who underwent both 3D-EUS and 3D-HRAM at a clinic specialised in proctology were included. A group of 30 patients without fistulas served as controls. Data regarding demographics, complaints, previous perianal surgical procedures and obstetric history were collected. The mean and regional anal pressures were measured with 3D-HRAM. Fistula tract areas detected with 3D-EUS were analysed with 3D-HRAM by visual coding and the regional pressures of the corresponding and surrounding area of the fistula tract areas were measured. The study was granted by the VUmc Medical Ethical Committee.

**Results:**

Forty patients (21 males, mean age 47) were included. Four patients had a primary fistula, 19 were previously treated with a seton/abscess drainage and 17 had a recurrence after previously performed fistula surgery. On 3D-HRAM, 24 (60%) fistula tract areas were good and 8 (20%) moderately visible. All but 7 (18%) patients had normal mean resting pressures. The mean resting pressure of the fistula tract area was significantly lower compared to the surrounding area (47 vs. 76 mmHg; *p* < 0.0001). Only 2 (5%) patients had a regional mean resting pressure < 10 mmHg of the fistula tract area. Using a Δ mean resting pressure ≥ 30 mmHg difference between fistula tract area and non-fistula tract area as alternative cut-off, 21 (53%) patients were identified. In 6 patients 3D-HRAM was repeated after surgery: a local pressure drop was detected in one patient after fistulotomy with increased complaints of faecal incontinence.

**Conclusions:**

Profound local anal pressure drops are found in the fistula tract areas in patients normal mean resting pressures. Fistulotomy may affect local sphincter pressure. This might influence surgical decision making in future.

## Background

Perianal fistulas as well as related surgical treatment may damage the anal sphincters leading to problems such as soiling and faecal incontinence with subsequent diminished quality of life. Especially recurrent fistulas are notorious [1–5]. Pre-operative identification of the fistula anatomy and sphincter function is important to reduce complications like recurrence or faecal incontinence. The current standard technique to visualise the anatomy of the anal sphincter complex is 3D endoanal ultrasonography (3D-EUS) or MRI. It provides information regarding the number of fistula tracts or sphincter defects, and thus guides surgical strategy [1, 2, 5–7]. According to a systematic review of the prevailing guidelines [8] and the Dutch Proctology Guidelines [9], a fistulotomy is performed in low (< 1/3 sphincter length) fistulas, while in higher (> 1/3 sphincter length) fistulas a more conservative approach is used by placing a seton or performing a sphincter saving technique such as a mucosal advancement plasty or a LIFT procedure [10]. Generally, a more conservative approach is performed in women with low anterior fistulas or in patients with concomitant sphincter damage [8, 9].

Anorectal manometry is used to evaluate functional anorectal disorders [11]. Previous studies have shown that fistulas and related surgery can lead to lower sphincter pressures resulting in faecal incontinence, although correlation between complaints and sphincter pressures has been reported to be poor [12–14].

Since the advent of 3D high-resolution anorectal manometry (3D-HRAM), regional pressures can be measured and a 3D reconstruction can be made, providing a pressure profile map [15]. Using 3D-HRAM we might be able to find regional differences in pressures, thus indicating whether the 3D-EUS defect is functional resulting in lower pressures, or should be considered as just a different ultrasound reflection. These findings could be used to tailor surgical therapy. This concept is indirectly supported by the studies by Vitton et al., who found only a moderate correlation between the detection of sphincter defects with 3D-HRAM and EUS [16, 17]. A recent study confirmed these results [18]. One study described focal defects as an area of absent pressure with surrounding areas of higher pressure; no comparison with another modality (EUS, MRI or surgery) was performed [19]. The inter-reader variability was fair for defects in this study.

With 3D-EUS an anatomical profile map can be obtained which has been shown to have impact on outcome of anal fistula surgery [20]. We postulate that information concerning regional pressures in patients with fistulas may play a future role in decision-making for fistula surgery. Will this make us more prudent in some patients with low fistulas to perform a fistulotomy and feel safe to perform a fistulotomy in some patients with higher fistulas? Therefore, we compared the 3D-HRAM pressure profile with the 3D-EUS anatomy in patients with perianal fistulas. The aim of the study was to address whether a fistula tract leads to local pressure abnormalities.

## Methods

This retrospective study was conducted at a clinic specialised in proctology. Consecutive patients diagnosed with active perianal fistulas between July 2014 and July 2017 who underwent both 3D-EUS and 3D-HRAM were included. All patients were treated according to the Dutch Proctology Guidelines [9]. 3D-EUS is a standard procedure in all patients with perianal fistulas. 3D-HRAM was performed on indication in patients in which a functional compromised sphincter complex needed to be excluded. A group of 30 patients (15 men and 15 women) without a fistula served as controls. Data was gathered regarding patients’ demographics, complaints, medical history, previous perianal surgical procedures, obstetric history and findings of the proctological examination at first presentation. All 3D-EUS and 3D-HRAM images were re-analysed together by two authors (RF and MV).

First we checked the 40 ultrasounds whether the fistula could be detected and the fistula was described. Secondly, the 3D-HRAM image was analysed to establish where areas of low pressure existed that could present a fistula or a pre-existing defect. This part was performed separately from the EUS analysis and thus blinded, results were noted. Finally, the EUS image was re-analysed and was compared to the 3D-HRAM, this part was not blinded. The study was granted by the VUmc Medical Ethical Committee.

### Three dimensional endoanal ultrasonography (3D-EUS)

A 3D-EUS system was used (Hawk type 2050, B-K Medical, Naerum, Denmark), with a rotating endoprobe with two crystals, covering 6–16 MHz (standard 12 MHz, focal range 2 to 4.5 cm, diameter 1.7 cm), producing a 360-degree view. 3D-EUS was performed by a proctologic surgeon (IHG, VRS, GVM, CDM) with the patient in the left lateral position, according to our previously described methods [1, 2]. The fistula tract was described as simple (one tract, low (< 1/3 sphincter length)), or complex (high (> 1/3 sphincter length) or branched), and also the tract course was analysed. A defect of the internal anal sphincter (IAS) was defined as a hyperechogenic interruption of the (hypoechogenic) muscular ring; a defect of the external anal sphincter (EAS) and puborectal muscle (PR) was defined as a hypoechogenic interruption of the muscle. The localisation of the sphincter defect was recorded as hours of the clock with 12 o’clock being anterior. Hydrogen peroxide 2% was used as a contrast agent to demonstrate fistula tracts.

### Three dimensional high resolution anorectal manometry (3D-HRAM)

**Fig. 1:**
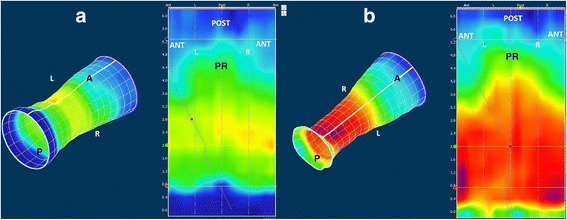
Normal 3D-HRAM. The 3D and 2D pressure profile in rest (**a**) and during contraction (**b**). Legend: The puborectal muscle (PR) muscle is seen in the middle of the 2D profile. ANT = anterior, POST = posterior, L = left and R = right. The white line in the cylinder is “cut” and unrolled to obtain the 2D image, so that the anterior is on both sides (ANT) of the 2D-image

The 3D-HRAM probe had 256 pressure sensors on 16 lines, each line having 16 circumferential sensors. The probe, which was covered by a disposable sheath, had a diameter of 10.75 mm, a length of 64 mm and an internal lumen to inflate the balloon (3.3 cm long with a capacity of 400 cm^3^). A specialised nurse performed the 3D-HRAM. All patients underwent the test in the left lateral position. No special bowel preparation was used. Pressures were measured at rest, during squeeze and during straining [16, 21]. Analysis of the manometry data was performed with ManoView (Given Imaging, Duluth, GA, USA). Presentation in both 2D and 3D images was used, and anal sphincter pressures in selected areas were measured using the smart mouse setting. To account for variation with the smartmouse measurements, we measured twice and used the average. The mean resting pressure (MRP) and mean squeeze pressure (MSP) were measured by the software. With ManoView the radial orientation of the 2D pressure reconstructions was determined by the pressure profile of the PR, shown as a longer pressure area posterior in relation to the shorter anterior pressure map. The middle of the PR pressure area was figured as the posterior part of the anal canal (Fig. 1)*.*

The anterior and posterior sphincter length were measured with the smartmouse function at 20 mmHg above rectal pressure as a threshold. The cursor was placed at the proximal part of the anal canal at the 20 mmHg level and a straight line was drawn to the distal part of the anal canal at again the 20 mmHg level. The sphincter length was based on the mean length of anterior and posterior pressure.

**Fig. 2:**
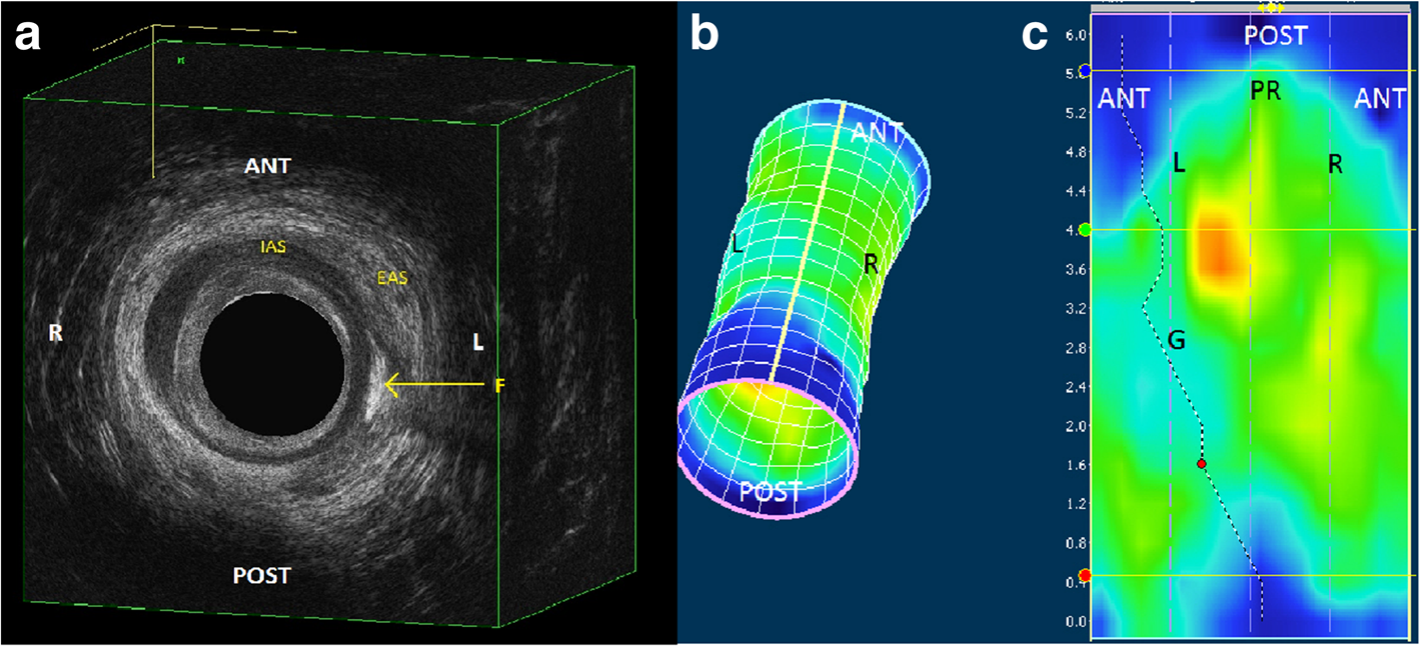
Comparison of 3D-HRAM with 3D-EUS in a patient with a fistula. Legend: The fistula tract visualised with 3D-EUS (**a**) is seen at 3 o’clock in the mid anal canal (on the left side of the patient) and corresponds very well with the pressure profile in (**b**) the 3D cylinder and (**c**) folded open 2D image. Note that the representation of the 3D-EUS is facing the patient’s anus, but with the 3D-HRAM left and right are switched, looking at the sphincter from behind. A low pressure “groove” (G) through the anal canal is also seen here. The dotted line is an automatic generated line of lowest pressure, and in this case that coincides with the groove. AS = internal anal sphincter, EAS = external anal sphincter, R = right, L = left, PR = puborectal muscle, ANT = anterior, POST = posterior

Areas of low pressure, judged upon the distinction of a different color, that could present a fistula or a preexisting defect pattern were noted. Longitudinal small sharp differences (ΔMRP ≥ 30 mmHg) throughout the sphincters shown in the pressure profile were defined as a groove (Fig. 2). Normal values of 3D-HRAM have been published in male and female adults by four different authors [21–24] and show a large range as well as an effect of gender and ageing. Therefore, we considered a MRP < 50 mmHg low since this was outside the 95% CI in these studies.

### Comparing 3D-HRAM with 3D-EUS

**Fig. 3:**
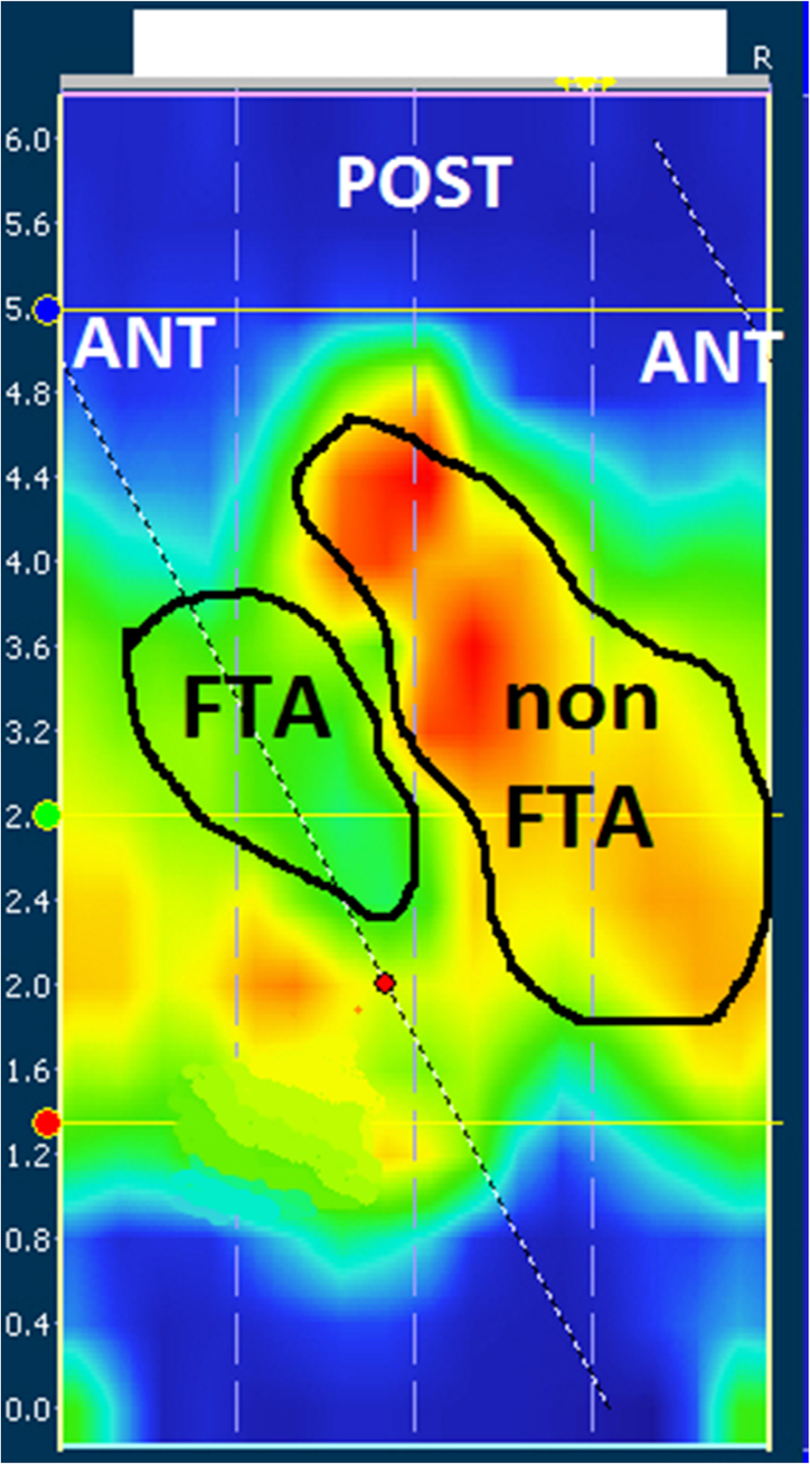
Measurement of difference between pressure in fistula tract area and the surrounding area. Legend: Smartmouse average measurements were made in both marked fistula tract area (FTA) and the non-fistula tract area (non-FTA)

The visual concordance of the fistula tract on 3D-EUS and same area the areas of low pressure with 3D-HRAM as mentioned above was scored as scored as good, moderate and poor. Where the 3D-EUS showed a fistula tract in the same area, the MRP and MSP in the fistula tract area (FTA), and the remaining area (non-FTA) was measured with the smart mouse function (Figs. 2 and 3). Two different ways to identify FTA area on 3D-HRAM were tested: 1. absolute local MRP in the FTA, 2. difference in resting pressure between the FTA and its surrounding area (ΔMRP). Subsequently, the MRP in the FTA was categorized as a MRP < 10 mmHg, MRP < 25 mmHg and a MRP < 50 mmHg for comparison with the literature [15–17]. Alternatively, we considered a ΔMRP of ≥30 mmHg as good concordance between 3D-HRAM and 3D-EUS.

### Statistical analysis

MRP and MSP were compared between the different groups using independent T-tests. For variables with more than two groups, analysis was done using one-way ANOVA, using Bonferroni or Games Howell as Post Hoc Test, depending on the equality of variances. The difference in pressure between FTA and non-FTA was tested using paired T-tests. Categorical variables were analysed using the Pearson Chi Square test. *P* ≤ 0.05 was considered as statistically significant (excepting Bonferroni correction). Analysis was performed using SPSS, version 23.

## Results

A total of 40 patients (21 males, 19 females) with a mean age of 47 years (range 24–69) were included. Four patients had a primary fistula, 19 were previously treated with a seton and/or abscess drainage and 17 had a recurrence after previous surgery (Table 1). Previously performed surgical procedures were fistulotomy (11), LIFT (8), mucosal advancement plasty (3) and laser therapy (1). Multiple operations were performed in 8 patients.

**Table 1:** 3D-EUS and 3D-HRAM findings in patients with fistulas

Patient groups	Total group	Primary fistula	Seton/abscess drainage	Recurrent fistula
Number of patients (Male/Female)	40 (21/19)	4 (3/1)	19 (5/14)	17 (13/4)
3D-EUS				
*Fistula type (n)*				
Simple	7	0	6	1
Complex	33	4	13	16
*Additional defects (n)*				
IAS	4 (2/2)	0	2 (0/2^a^)	2 (2/0)
EAS	4 (1/3)	0	2 (0/1^a^1)	2 (1/1)
IAS + EAS	4 (3/1)	0	1 (1/0)	3 (2/1^a^)
3D-HRAM				
MRP (mmHg)	69	81	65	70
MSP (mmHg)	125	145	106	143
MRP FTA (mmHg)	47	66	42	47
MRP Non-FTA (mmHg)	76	95	74	73
MSP FTA (mmHg)	92	111	82	99
MSP Non-FTA (mmHg)	138	175	130	138
Sphincter length (cm)	3.3	4.4	3.2	3.0
Groove (*n*)	11	2	5	4
Simple fistula	0	0	0	0
Complex fistula	11	2	5	4
Agreement 3D-EUS and 3D-HRAM				
Good	24	3	12	9
Moderate	8	0	4	4
No	8	1	3	4

^a^Women with a traumatic delivery. *IAS* internal anal sphincter, *EAS* external anal sphincter, *MRP* mean resting pressure, *MSP* mean squeeze pressure, *FTA* fistula tract area

Fourteen out of 19 (74%) women had had one or more vaginal deliveries (VD), of whom 12 had a traumatic delivery (perineal tear or episiotomy).

In addition to their complaints of the perianal fistula, two patients were incontinent for liquid faeces. One patient was a 37-year old female with an anterior high transsphincteric fistula and a traumatic delivery, and the other patient was a 66-year old male with a high transsphincteric fistula and three previous fistula surgical interventions, including resection of a sinus in the sphincter. Their mean MRP and MSP were within the normal range.

The data of the control patients are shown in Table 2. Diagnoses of the patients were faecal incontinence (11), soiling (5), anal fissures (5), constipation (3), pelvic pain (3), other (3) (enterostoma [2], Hirschsprung’s disease [1]).

**Table 2:** 3D-EUS and 3D-HRAM findings in 30 control patients

Patient groups	Defects on EUS	No defects on EUS
Number of patients (Male/Female)	10 (5/5)	20 (10/10)
3D-EUS		
*Defects*		
IAS	5 (5/0)	-
EAS	4 (0/4^a^)	-
IAS + EAS	1 (0/1^a^)	-
3D-HRAM		
MRP (mmHg)	65	75
MSP (mmHg)	93	125
MRP defect (mmHg)	27	–
MRP non-defect (mmHg)	68	–
MSP defect (mmHg)	52	–
MSP non-defect (mmHg)	100	–
Sphincter length (cm)	3.1	3.2
Groove (*n*)	6 (3/3)	–
Agreement 3D-EUS and 3D-HRAM		
Good	6 (3/3^a^)	–
Moderate	4 (2/2^a^)	–
No	0	–

^a^Women with a traumatic delivery. *IAS* internal anal sphincter, *EAS* external anal sphincter, *MRP* mean resting pressure, *MSP* mean squeeze pressure

### 3D-EUS

#### Fistula tracts

The fistula tract was identified on 3D-EUS in all 40 patients (Table 1). Seven patients had a simple fistula and 33 patients a complex fistula. Side branches were present in 5 patients. Besides the fistula tract, additional sphincter defects were seen in 12 (30%) of the patients. Of these patients, 7 had previously performed fistula related surgery, two had high fistulas with abscess/seton drainage and three women had had a traumatic delivery and previous treatment with a seton.

### 3D-HRAM

The results of the 3D-HRAM are shown in Table 1. In 7 patients (18%) a MRP < 50 mmHg was found. Five were females who all had had traumatic VDs, three had simple fistula and two had complex fistulas. Both men had recurrent suprasphincteric fistulas.

There were no differences in pressures or sphincter length between patients with or without previous treatments or recurrences, but since men were over represented in the recurrent group no conclusion could be made.

In 11 (28%) patients a groove was seen with 3D-HRAM, all were complex fistulas.

In 6 patients (all with a previous seton/abscess drainage, one mucosal advancement plasty, one excision sinus) a second 3D-HRAM was performed after surgery. The surgical procedures were single or combined procedures: seton (2), LIFT (2) Permacol (2) abscess drainage with mucosal advancement plasty (2) and fistulotomy (3). The mean pressures did not change after these procedures. However, in the patient with a previous excision of a sinus, a distinct distal local pressure drop became evident after fistulotomy. The 3D-EUS showed disappearance of the fistula and healing with a scar. The patient’s complaints of faecal incontinence increased.

### Comparing 3D-HRAM with 3D-EUS

#### 3D-HRAM pressures in 3D-EUS FTA compared to non-FTA

The MRP in the FTA was significantly lower (47 mmHg, SD 23) compared to the non-FTA (76 mmHg, SD 21) (*p* < 0.0001). The MSP was also significantly lower in the FTA (92 mmHg, SD 48) compared to the non-FTA (138 mmHg, SD 51) (*p* < 0.0001).

#### Absolute MRP in the FTA

Two patients (5%) had a regional MRP < 10 mmHg in the FTA [15, 16], 6 (15%) patients had regional MRP < 25 mmHg [17] and 21 (53%) patients had a regional MRP ≤ 50 mmHg.

#### ΔMRP between FTA and non-FTA

The fistula tract was visible in 32 (80%). The visual score was good in 24 (60%) and moderate in 8 (20%) patients (Table 1). The mean ΔMRP was 29 mmHg (SD 19). A ΔMRP of ≥30 mmHg was found in 21 (53%) patients. Of the 33 (83%) patients with a normal MRP (> 50 mmHg), 17 (52%) had a ΔMRP of ≥30 mmHg between the FTA and the surrounded area.

### Control group

Five (of 30) patients (17%) had a MRP < 50; 3 had faecal incontinence, one rectal prolapse and one Hirschsprung’s disease. Patients with faecal incontinence had a lower MRP than patients with fissures or soiling (59, 90 and 80 mmHg respectively; *p* = 0.03 between faecal incontinence and fissures) and MSP (62, 121 and 122 respectively; no significant difference).

There were 10 patients with a defect on anal ultrasound. Three patients (30%) had a regional MRP < 10 mmHg in the defect area, 6 (60%) patients had regional MRP < 25 mmHg. A ΔMRP of ≥30 mmHg was found in 8 (80%) patients.

The concordance between 3D-EUS and 3D-HRAM was good in 6 and moderate in 4 patients. Those with good concordance had a childbirth injury (3) and anorectal surgery (hemorrhoidectomy 2, lateral internal sphincterotomy 1); all defects throughout the length of the sphincter and had a groove. The four with moderate concordance had only internal sphincter defects from a deep fissure, and Hirschsprung’s disease (2), or a small external sphincter defect from childbirth (2). These were small areas of lower pressure and there were no grooves. In patients without defects on 3D-EUS, some variation in pressures was seen in the 2D and 3D patterns comparable to a “normal” 3D-HRAM (Fig. 1) and no grooves were found.

## Discussion

This study has shown that there is a moderate to good visual agreement between the fistula tract on 3D-EUS and a 3D-HRAM pressure drop in the same area in patients with active perianal fistulas. Measurements showed significant differences: the mean differences between the FTA and non-FTA was 29 mmHg in MRP and 46 mmHg in MSP. In the control patients who had a sphincter defect, similar differences in pressure were found.

When measuring actual pressures in both areas, it becomes difficult to define a pressure abnormality and translate this into a threshold pressure. This issue has been addressed by Vitton in patients with sphincter defects [16]. She defined a defect in the IAS or EAS as a MRP or MSP of < 10 mmHg respectively; the detection rates were 59% and 56% respectively for the defects found with EUS. False positive rates, suggesting a defect with 3D-HRAM not confirmed by EUS were 21% and 30%. Automatic analysing software did not improve the detection rate [17]. Another study in 39 patients with a threshold of 25 mmHg with 18^o^ continuous expansion showed that sphincter defects on 3D-EUS were detected with 3D-HRAM in 6 out of 8 patients (75%) [18]. Furthermore, in the 23 patients without a defect on 3D-EUS, a defect was suggested with 3D-HRAM in 8 patients. These settings improved the detection rate, however the number of detected sphincter defects was low (21%).

In our patients with FTA using MBP < 10 mmHg we only detected 5%, a threshold of 25 mmHg increased that to 15% of the FTA found on 3D-EUS. This suggests that not all ultrasound abnormalities are functional and low pressures cannot always be seen with 3D-EUS. We postulate that an alternative method might be the difference in MRP between the FTA and the non-FTA, in which a difference of ≥30 mmHg could be a better determinant. Using this method the detection rate improved to 53% which is unfortunately still moderate. The results in the control patients with sphincter defects were similar.

Remarkable was the observed large variance in individual pressure differences between the FTA and the surrounding area, varying from 0 mmHg up to 70 mmHg. Still 47% of the population had a pressure difference < 30 mmHg, therefore a FTA would be missed when defined as such. Further lowering of the definition of difference could give false positive defects in patients, as physiological differences in pressure would be falsely identified as FTA. Clearly, 3D-HRAM measures function and the fistula tract only becomes visible if it leads to pressure drops.

More interesting are the additional findings provided by 3D-HRAM. In 33 (83%) patients a normal MRP was found. Of these, 17 (52%) had profound low local pressure drop of ≥30 mmHg in their FTA. In some pressure profiles these areas were visible as a groove (Fig. 2c): long small differences in pressure profile. In the control patients grooves were found in those with large defects throughout the sphincter. The groove aspect seems to be related to large complex fistulas or large sphincter defects.

In 6 patients a second 3D-HRAM was performed after subsequent surgery. In one male patient with a recurrent fistula the 3D-HRAM showed a drop of the local pressure profile without a change in mean pressure. The repeated 3D-EUS showed disappearance of the fistula tract and scar tissue. His complaints of faecal incontinence increased.

These are interesting findings, but what do they mean? Most surgeons will consider normal mean anal pressures as a permit to perform a fistulotomy. Are the patients with normal mean anal pressures but with large local pressure drops prone to develop postoperative faecal incontinence? How low may the local pressure drop be before becoming important as a risk of postoperative faecal incontinence? Or is it only of importance in women after VD or patients who underwent multiple surgical procedures? At this point 3D-HRAM does not provide clear answers to these questions. Prospective evaluation including questionnaires, 3D-HRAM and 3D-EUS in these patients before and after fistulotomy is necessary. This study aimed to evaluate the pressure profiles in relation to the fistula tracts.

There are some limitations to this study. First, normal values with 3D-HRAM have not uniformly been established. To date, normal values have been reported in 6 studies, four in healthy adults [21–25], one in pregnant women [26] and one in children [27]. Reproducibility of 3D-HRAM has been assessed in two studies showing a good to moderate correlation [22, 28]. 3D-HRAM cannot be directly compared with flexible thinner HRAM catheters, with which more studies on normal values have been performed [29, 30]. Furthermore, 3D-HRAM has been demonstrated to measure higher pressures than conventional anal manometry [31]. In addition, ageing and female sex are correlated with lower anal pressures and a large overlap between normal controls and patients has been shown in previous studies [13, 16, 17, 21–24, 28]. In this study we used > 50 mmHg as a normal MRP since this was inside the 95% CI in all studies. Other limitations of this study are the relatively small patient groups and the retrospective nature of the study.

In spite of these shortcomings, the results warrant further prospective studies in patients with perianal fistulas before and after surgery to gain more insight with possible clinical consequences. Considering the costs of 3D-HRAM we cannot recommend this as a routine procedure in patients undergoing fistula surgery use at this point.

## Conclusions

Fistula tracts result in lower local pressures seen with 3D-HRAM. Detection by means of a threshold pressure is not feasible. Patients had generally normal average anal pressures, but 3D-HRAM showed profound local pressure drops in about 53% of patients, which sometimes had the aspect of a groove. Fistulotomy can cause local pressure drops. The extent of these pressure drops after surgery may be interesting to evaluate prospectively in patients to determine whether they correlate with functional outcome and it may influence type of fistula surgery if added to 3D-EUS findings.

## Abbreviations

3D-EUS: 3D endoanal ultrasonography
3D-HRAM: 3D high resolution anorectal manometry
EAS: External anal sphincter
FTA: Fistula tract area
IAS: Internal anal sphincter
MRP: Mean resting pressure
MSP: Mean squeeze pressure
PR: Puborectal muscle
